# The Nature of General Shields’ Wound

**Published:** 1848-04

**Authors:** 


*The Nature of General Shields' Wound.*—This gallant soldier has recently been the guest of our city, and we were called upon to dress his second wound: being detained, we found our friend, Dr. Dugas, in attendance when we arrived. It is known that Gen. Shields was wounded twice in the recent battles in Mexico. By the discharge of a *cannon* at Cerro Gordo, he was shot through the body and given over as certain to die. The General thinks it was a grape shot that traversed his chest. The balls has evidently passed between the *lungs, through the mediastina;* entering within the right nipple, and passing out near the spine on the right side. He spat no blood, did not fall, and even gave the word of command after being wounded. In a few moments he was in indescribable agony, and even prayed for death, to be relieved!

None but a medical man can fully appreciate the nature of this wound, which has no parallel on record.—*Southern Medical and Surgical Journal.*

